# A Secure and Verifiable Outsourced Access Control Scheme in Fog-Cloud Computing

**DOI:** 10.3390/s17071695

**Published:** 2017-07-24

**Authors:** Kai Fan, Junxiong Wang, Xin Wang, Hui Li, Yintang Yang

**Affiliations:** 1 State Key Laboratory of Integrated Service Networks, Xidian University, Xi’an 710071, China; wangjx921210@163.com (J.W.); xwang2011@stu.xidian.edu.cn (X.W.); lihui@mail.xidian.edu.cn (H.L.); 2 Key Laboratory of the Ministry of Education for Wide Band-Gap Semiconductor Materials and Devices, Xidian University, Xi’an 710071, China; ytyang@xidian.edu.cn

**Keywords:** fog computing, cloud computing, access control, attribute-based encryption, verifiable outsource, revocation

## Abstract

With the rapid development of big data and Internet of things (IOT), the number of networking devices and data volume are increasing dramatically. Fog computing, which extends cloud computing to the edge of the network can effectively solve the bottleneck problems of data transmission and data storage. However, security and privacy challenges are also arising in the fog-cloud computing environment. Ciphertext-policy attribute-based encryption (CP-ABE) can be adopted to realize data access control in fog-cloud computing systems. In this paper, we propose a verifiable outsourced multi-authority access control scheme, named VO-MAACS. In our construction, most encryption and decryption computations are outsourced to fog devices and the computation results can be verified by using our verification method. Meanwhile, to address the revocation issue, we design an efficient user and attribute revocation method for it. Finally, analysis and simulation results show that our scheme is both secure and highly efficient.

## 1. Introduction

Recently, fog computing has drawn a great deal of attention. It is a quite novel computing paradigm that extends cloud computing facilities and services to the edge of the network to provide computing, networking, and storage services between end devices and data centers [1,2]. Fog computing devices are located between endpoints and the traditional cloud, thus resources and services are available and are closer to the end-users, and the delays induced by service deployments can be reduced [3,4]. Compared with the cloud computing concept, which is more centralized, fog computing provides resources and services in a distributed way. Combined with the traditional cloud, faster and more convenient computing services are provided to nearby devices based on their own computing, storage and network capacity [5]. Since fog devices are localized, it provides low-latency communication and more context awareness [6]. With all these advantages, the fog computing paradigm is well positioned for big data and real time analytics.

Fog computing is a quite novel computing paradigm that aims at moving the cloud computing (CC) facilities and services to the access network, in order to reduce the delays induced by service deployments. Although big data and the Internet of things (IOT) still rely on cloud computing, as the number of networking devices and data volume are increasing dramatically, fog-cloud computing can effectively solve the bottleneck problem of data transmission and data storage. However, since fog devices are located at the edge of the network and are of much lower cost than cloud servers, they are more easily compromised and have lower trustworthiness [7,8], especially in the process of data sharing. Therefore, secure and efficient access control schemes in fog-cloud computing environment need to be implemented [9,10]. Compared with traditional data access control schemes in cloud computing, the network structures and system models in the fog-cloud computing environment are different. Fog devices can provide computing, networking, and storage services for users, such that less communication and computational cost is left for users to do, therefore, cloud, fog and end-users should be considered in the new access control scheme.

Ciphertext-policy attribute-based encryption (CP-ABE) [11] is regarded as one of the most suitable technologies to realize fine-grained access control. This technique allows data owners to implement access control by setting up access structures. Compared with single-authority CP-ABE schemes, in multi-authority CP-ABE schemes, attributes are from different domains and managed by different authorities. Moreover, it does not have the single point of failure and system bottleneck problem, which makes multi-authority CP-ABE schemes more practical for data access control in the fog-cloud computing environment.

However, the processes of encryption and decryption in CP-ABE systems are time-consuming. The computation for data owners and users is a great overhead. To outsource part of the encryption and decryption computation to a cloud server is a solution. However, the server may be “lazy”. It may not follow the algorithm, and only execute part of the computations or deliberately return incorrect results. Therefore, a verification method of the outsourced encryption and decryption needs to be proposed. Besides, user and attribute revocation is another issue in CP-ABE systems. On the one hand, the users in the system may change frequently, and on the other hand, the attributes of users may also change, and revocation of any attribute may affect other users who share the same attribute. However, most existing schemes cannot support flexible and efficient user and attribute revocation in multi-authority cloud storage systems. The key update and ciphertext re-encryption operations are time-consuming. Therefore, verifiable outsourced multi-authority CP-ABE schemes with efficient and flexible user and attribute revocation need to be proposed.

### 1.1. Related Work

In 2007, Bethencourt et al. [11] put forward the first CP-ABE scheme. Over the last decade, many CP-ABE schemes [12,13,14,15,16,17] were proposed. However, most of them are time-consuming and lack efficiency. To improve the efficiency and reduce the overhead of users, several schemes which support outsourced computation and revocation are proposed:

#### 1.1.1. Outsourced Computation 

Green et al. [18] proposed an outsourcing decryption ABE scheme. In their scheme, the traditional private keys are divided into user keys and transformation keys. Thus, complex decryption computations are outsourced to the cloud server, and users only need one exponentiation operation to recover the plaintext. However, their scheme cannot be applied to multi-authority systems. Based on this method, Yang et al. [19,20] put forward two multi-authority CP-ABE schemes which support outsourced decryption. Li et al. [21] also proposed an outsourced ABE scheme which supports both outsourced key-issuing and decryption. However, they did not consider the correctness of results from the cloud server.

To solve this problem, Lai et al. [22] introduced the verifiability of ABE and proposed a verifiable outsourced decryption ABE scheme. But in their scheme, both the length of the ciphertext and the computational of encryption are doubled. Later, Li et al. [23] presented an outsourcing ABE scheme with checkability which supports both outsourced key-issuing and decryption. However, the length of ciphertext and the amount of expensive pairing computations grow with the number of attributes. 

To address this problem, two ABE schemes [24,25] in which the length of ciphertext is constant are put forward. However their constructions cannot be applied to ABE schemes with Linear Secret Sharing Schemes (LSSS). Mao et al. [26] proposed a generic construct of attribute-based encryption with verifiable outsourced decryption. Their CPA-secure construct has more compact ciphertext and less computational costs. Users only need a constant number of simple computations to decrypt the ciphertext.

#### 1.1.2. Revocation 

Ostrovsky et al. [27] first proposed a fine-grained user revocation scheme based on CP-ABE that supports negative clauses. With the help of a semi-trusted service provider, Ibraimi et al. [28] put forward a CP-ABE scheme which achieved immediate attribute revocation for the first time, but their construct cannot be applied to an outsourcing environment. Yu et al. [29] presented a CP-ABE scheme where proxy encryption technology was introduced. The scheme achieves immediate attribute revocation, at the same time, the proxy server also share the authority job, hoowever, the proxy server needs to be online all the time. Another CP-ABE scheme with fine-grained attribute revocation was put forward by Hur et al. [30]. They use attribute group keys to re-encrypt the ciphertext, but their scheme cannot prevent collusion attacks. Another revocable CP-ABE scheme was proposed by Xie et al. [31]. In their scheme, the key update computations are greatly reduced. Later, Yang et al. [32] put forward a proxy-assisted CP-ABE scheme which provides efficient cloud data sharing and user revocation.

### 1.2. Our Contribution

In this paper, we propose a verifiable outsourced multi-authority access control scheme, named VO-MAACS. In our construct, most of the encryption and decryption computation is outsourced to fog devices and the computation results can be verified by using our verification method. Meanwhile, to address the revocation issue, we design an efficient user and attribute revocation method for it. Our contributions can be summarized as follows:

(1) We propose the verifiable outsourced multi-authority access control scheme (VO-MAACS), which is secure against collusion attacks. Most of the encryption and decryption computation is outsourced to fog devices, which greatly reduces the computation on the user side.
(2) We provide a verification method for the outsourced encryption and decryption. If a fog device returns incorrect results, users can notice it immediately by running the corresponding verification algorithm.
(3) We design an efficient user and attribute revocation method for our scheme. During the process of attribute revocation, most of the update and re-encryption operations are outsourced to the cloud server, and only a few components which are associated with the revoked attribute need to be updated, while the other components are not changed.
(4) We provide a security and performance analysis of our scheme, which shows that our scheme is both secure and highly efficient.

### 1.3. Organization

The remainder of this paper is organized as follows: we first give some preliminaries in Section 2. Then, we give the definition of the system model and framework in Section 3. In Section 4, we propose our VO-MAACS construct. Section 5 describes the security and performance analysis of our scheme. Finally, the conclusions are given in Section 6.

## 2. Preliminaries 

In this section, some fundamental background used in this paper is provided, including bilinear maps, access structure and linear secret sharing scheme (LSSS).

### 2.1. Bilinear Maps

**Definition 1. (Bilinear Maps).**  *Let* $G_{1}$, $G_{2}$ *and* $G_{T}$ *be three cyclic groups of prime order* p. *A bilinear map is a map* $e:G_{1}\times G_{2}\to G_{T}$ *with the following properties:*

*(1)*  Bilinearity: for all $g_{1}\in G_{1}$, $g_{2}\in G_{2}$ and $a,b\in Z_{p}$, $e(g_{1}^{a},g_{2}^{b})=e{\left(g_{1},g_{2}\right)}^{ab}$.
*(2)*  Non-degeneracy: there exists $g_{1}\in G_{1}$, $g_{2}\in G_{2}$ such that $e(g_{1},g_{2})=1$.
*(3)*  Computability: there is an efficient algorithm to compute $e(g_{1},g_{2})$ for any $g_{1}\in G_{1}$ and $g_{2}\in G_{2}$.

### 2.2. Access Structure

**Definition 2. (Access Structure [33]).**  *Let* $\left\{P_{1},P_{2},\cdots ,P_{n}\right\}$ *be a set of parties. A collection* $A\subseteq 2^{\left\{P_{1},P_{2},\cdots ,P_{n}\right\}}$ *is monotone if* ∀B,C*: if* B∈A *and* B⊆C *then* C∈A*. An access structure (respectively, monotone access structure) is a collection (respectively, monotone collection)* A *of non-empty subsets of* $\left\{P_{1},P_{2},\cdots ,P_{n}\right\}$*, i.e.,* $A\subseteq 2^{\left\{P_{1},P_{2},\cdots ,P_{n}\right\}}\backslash \{\phi \}$*. The sets in* A *are called the authorized sets, and the sets not in* A *are called the unauthorized sets.*

### 2.3. Linear Secret Sharing Schemes

**Definition 3. (Linear Secret-Sharing Schemes (LSSS) [33]).**  *We recall the description of LSSS as follows [33]. Let* Π *be a secret sharing scheme over a set of parties* P *with realizing an access structure* A. *We say that* Π *is a linear secret sharing scheme over* $Z_{p}$ *if:*

*(1)*  The piece for each party forms a vector over $Z_{p}$.
*(2)*  *During the generation of the pieces, the dealer chooses independent random variables, denoted* $r_{2},\cdots ,r_{n}$, each one distributed uniformly over $Z_{p}$. *Each coordinate of the piece of every party is a linear combination of* $r_{2},\cdots ,r_{n}$ *and the secret* s. *That is, let* M *denotes a matrix with* l *rows and* n *columns. For the vector* ${\vec{v}}^{T}=(s,r_{2},\cdots ,r_{n})$ *and any authorized set, there exist constants* ${\left\{w_{i}\in Z_{p}\right\}}_{i\in I}$ *such that, if* $\left\{\lambda_{i}\right\}$ *are valid shares of any secret* s *according to* Π, *then* $\sum_{i\in I}w_{i}\lambda_{i}=s$, *where* $\lambda_{i}={\left(M\vec{v}\right)}_{i}$ *and* I⊂{1,2,⋯,l}.

## 3. System Model and Framework

In this section, the system model and framework of our scheme are described.

### 3.1. System Model

**Figure 1:**
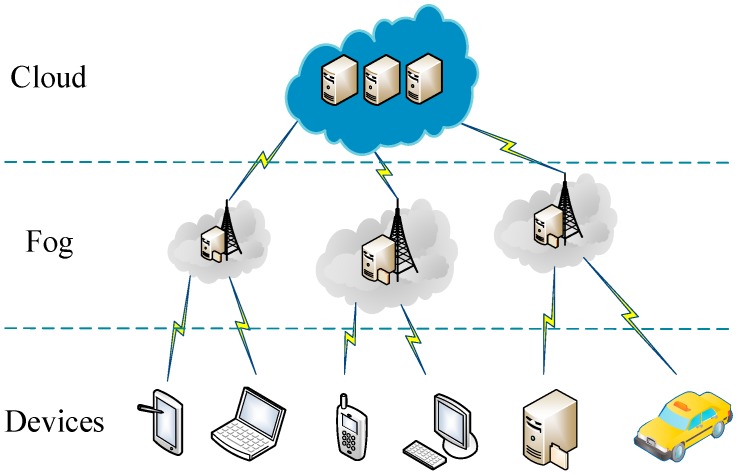
System model in fog-cloud computing environment.

A simple three level hierarchy is adopted in our fog-cloud system as illustrated in Figure 1. In this framework, each terminal device is connected to a nearby fog device. Fog devices are interconnected and each of them is linked to the cloud.

In general, a layer of fog is added between the cloud server and terminal devices so that some computations on the cloud server can be delegated to the fog devices which are closer to the terminal devices. Thus, different tasks from different regions can be executed by the corresponding fog devices simultaneously, which greatly improves the efficiency. Fog devices are responsible for data transmission and data storage. Moreover, they are also in charge of part of the encryption and decryption computations. The cloud server is responsible for storing the ciphertext and the user proxy keys, as well as the ciphertext re-encryption operations and user proxy keys update operations when revocation occurs.

**Figure 2:**
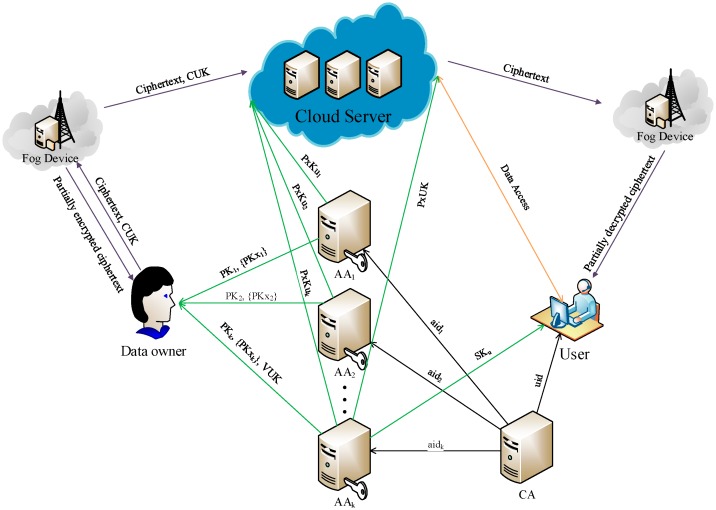
System model of multi-authority access control in fog-cloud system.

Our multi-authority fog-cloud system consists of six entities: a cloud service provider (CSP), fog devices (FDs), a global certificate authority (CA), attribute authorities (AAs), data owners (DOs) and data users (DUs), as shown in Figure 2.

CA is a fully trusted global certificate authority in the system. It accepts the registration of all AAs and DUs in the system, and it is responsible for issuing a global unique identity uid for each DU and a unique identity aid for each AA. However, it does not participate in any attribute management and any generation of secret keys associated with attributes. 

Each AA is an independent attribute authority that is responsible for issuing, revoking and updating users’ attributes within its administration domain. In our scheme, each AA is responsible for generating a public attribute key $PK_{x}$ for each attribute it manages and a user private key which consists of user proxy key PxK and user secret key SK for each DU. Especially, PxK is stored at CSP and SK is kept by DU.

DOs define access control policies over attributes from multiple attribute authorities and then encrypts the data following those policies. After that, they upload the encrypted data to the CSP.

The CSP is responsible for storing the ciphertext and the user proxy keys, and provides data access service to DUs. It is also in charge of the ciphertext re-encryption operations and user proxy key update operations when revocation occurs.

FDs are responsible for data transmission and data storage. Moreover, they are also in charge of part of the encryption and decryption computations. They can help generate part of the ciphertext for DOs, as well as decrypt part of the ciphertext for DUs. Only for those DUs whose attributes satisfy the access policy will FDs decrypt the ciphertext with their proxy keys. After that, they send the partially decrypted data to the corresponding DUs.

DUs can request their secret keys from the relevant authorities. After downloading any encrypted data from the CSP, a DU first asks a FD to decrypt it with his proxy key. If the attribute set of the DU meets the access policy, then the FD decrypts the ciphertext and sends the partially decrypted data to the DU. Upon receiving the partially decrypted data from the FD, the DU can recover the data with his secret key.

In our multi-authority fog-cloud system, we assume that the CA is fully trusted in the system. Each AA is also trusted, but it can be corrupted by an adversary. The CSP and FDs are semi-trusted. They may leak the encrypted data to some malicious users, but will execute the tasks assigned by each authority. DUs are assumed dishonest and may collude to obtain unauthorized access to data.

### 3.2. Framework

**Definition 4. (VO-MAACS: Verifiable Outsourced Multi-Authority Access Control Scheme).**  ***Global Setup*** (λ,U)→{GP,uid,aid}. *The global setup algorithm is run by CA. On input the security parameter* λ *and attribute universe description* U, *it outputs the global parameter* GP*, the user identity* uid *and the authority identity* aid.

***Authority Setup*** $\left(aid)\to \{PK_{aid},SK_{aid},{\left\{PK_{x_{k}}\right\}}_{aid\in I_{A}}\right\}$*. The authority setup algorithm is run by each authority. On input of the authority identity aid, it outputs public attribute keys ${\left\{PK_{x_{k}}\right\}}_{aid\in I_{A}}$ for all attributes issued by each authority and a pair of authority public key $PK_{aid}$ and authority secret key $SK_{aid}$. Here $I_{A}$ denotes the involved authority set.*

***Encrypt_out*** $(GP,{\left\{PK_{x_{k}}\right\}}_{aid\in I_{A}})\to CT_{out}$*. The outsourced encryption algorithm is run by the FD. On input of the global parameter* GP *and a set of public attribute keys* ${\left\{PK_{x_{k}}\right\}}_{aid\in I_{A}}$*, it outputs the partially encrypted ciphertext $CT_{out}$.*

***Verify_enc*** $(GP,CT_{out})\to b$*. The outsourced encryption verification algorithm is run by a DO. On input of the global parameter* GP *and a partially encrypted ciphertext* $CT_{out}$*, it outputs a bit b∈{0,1}, b=1 indicates the FD outputs the correct result,* b=0 *indicates the FD outputs the incorrect result*.

***Encrypt_user*** $(GP,PK_{aid},{\left\{PK_{x_{k}}\right\}}_{aid\in I_{A}},CT_{out},M,(A,\rho ))\to CT$*. The user encryption algorithm is run by a DO. On input of the global parameter* GP*, a set of authority public keys* $PK_{aid}$*, a set of public attribute keys* ${\left\{PK_{x_{k}}\right\}}_{aid\in I_{A}}$*, a partially encrypted ciphertext* $CT_{out}$*, a message* M *and an access structure* A*, it outputs the ciphertext CT.*

***KeyGen*** $(GP,uid,S_{uid,aid},SK_{aid},{\left\{PK_{x_{k}}\right\}}_{aid\in I_{A}})\to \left\{PxK_{uid,aid},SK_{uid}\right\}$*. The key generation algorithm is run by each authority. On input of the global parameter* GP*, the user identity* uid*, a set of user attributes* $S_{uid,aid}$*, the authority secret key* $SK_{aid}$*, and a set of attribute public keys* ${\left\{PK_{x_{k}}\right\}}_{aid\in I_{A}}$*, it outputs user proxy key* $PxK_{uid,aid}$ *and user secret key $SK_{uid}$.*

***Decrypt_out*** $(GP,CT,PxK_{uid,aid},{\left\{PK_{x_{k}}\right\}}_{aid\in I_{A}})\to CT^{\prime }$*. The outsourced decryption algorithm is run by a FD. On input of the global parameter* GP*, the proxy keys* $PxK_{uid,aid}$ *and the ciphertext* CT*, it outputs the partially decrypted ciphertext $CT^{\prime }$.*

***Verify_dec*** $(GP,CT,CT^{\prime })\to b$*. The outsourced decryption verification algorithm is run by a DU. On input of the global parameter* GP*, the ciphertext* CT *and a partially decrypted ciphertext* $CT^{\prime }$*, it outputs a bit b∈{0,1}, b=1 indicates the FD has output the correct result, b=0 indicates the FD has output the incorrect result.*

***Decrypt_user*** $(CT,CT^{\prime },SK_{uid})\to M$. *The user decryption algorithm is run by a DU. On input of the ciphertext CT, the partially decrypted ciphertext $CT^{\prime }$ and the user secret key $SK_{uid}$, it outputs the message M.*

***URev*** $(uid,L_{PxK})\to {L^{\prime }}_{PxK}$*. The user revocation algorithm is run by the CSP. On input of the revoked user identity* uid *and the proxy key list $L_{PxK}$, it outputs the updated proxy key list ${L^{\prime }}_{PxK}$.*

***ReKeyUpdate*** $\left(uid,PxK_{uid,aid},v_{{\tilde{x}}_{k}})\to \{VUK_{{\tilde{x}}_{k}},PxUK_{{\tilde{x}}_{k}}\right\}$*. The key update algorithm is run by the involved authorities. On input of the* uid *of each non-revoked user, the proxy key* $PxK_{uid,aid}$ *and the current attribute version key* $v_{{\tilde{x}}_{k}}$*, it outputs the version update key* $VUK_{{\tilde{x}}_{k}}$ *and the proxy update key $PxUK_{{\tilde{x}}_{k}}$.*

***CTUpdate*** $\left(VUK_{{\tilde{x}}_{k}},CT_{out})\to \{CUK_{{\tilde{x}}_{k}}\right\}$*. The ciphertext update algorithm is run by a DO. On input of the version update key* $VUK_{{\tilde{x}}_{k}}$ *and the partially encrypted ciphertext* $CT_{out}$*, it outputs the ciphertext update key $CUK_{{\tilde{x}}_{k}}$.*

***PxKUpdate*** $(uid,PxK_{uid,aid},PxUK_{{\tilde{x}}_{k}})\to PxK_{uid,aid}^{*}$. The proxy key update algorithm is run by the CSP. On input of the uid *of each non-revoked user, the current proxy key* $PxK_{uid,aid}$ *and the proxy update key* $PxUK_{{\tilde{x}}_{k}}$*, it outputs a new proxy key* $PxK_{uid,aid}^{*}$ *for each non-revoked user who has the attribute ${\tilde{x}}_{k}$.*

***ReEnc*** $(CT,CUK_{{\tilde{x}}_{k}})\to CT^{*}$. The re-encryption algorithm is run by the CSP. On input of the current ciphertext CT *and the ciphertext update key* $CUK_{{\tilde{x}}_{k}}$, it outputs a new ciphertext $CT^{*}$.

## 4. VO-MAACS: Verifiable Outsourced Multi-Authority Access Control Scheme

In this section, we give the concrete construction of VO-MAACS which is based on [14], together with the verification method and revocation scheme. 

### 4.1. Construction of VO-MAACS

*Global Setup* (λ,U)→{GP,uid,aid}. The global setup algorithm takes a security parameter λ and a small attribute universe description U as input. Let $G_{1}$, $G_{2}$ and $G_{T}$ be the multiplicative groups with the same prime order p, and $e:G_{1}\times G_{2}\to G_{T}$ be the bilinear map. Let $g_{1}$ be the generator of $G_{1}$ and $g_{2}$ be the generator of $G_{2}$. Let $G:G_{T}\to Z_{p}$ be a hash function and $H:{\left\{0,1\right\}}^{*}\to Z_{p}$ be a hash function which maps attributes to an element in $G_{2}$, such that the security will be modeled in the random oracle. CA then chooses a random number $a\in Z_{p}$ and sets the global parameter as $GP=\{p,G_{1},G_{2},G_{T},e,g_{1},g_{2},g_{2}^{a},H,G\}$. Each authority, fog device and user should register itself with the global authority during the global setup process. CA then assigns a unique global authority identity aid to each legitimate authority and a unique global user identity uid to each legitimate user.

*Authority Setup* $\left(aid)\to \{PK_{aid},SK_{aid},{\left\{PK_{x_{k}}\right\}}_{aid\in I_{A}}\right\}$. Let $S_{A_{aid}}$ denote the set of all attributes managed by $AA_{aid}$ and $I_{A}$ denote the involved authority set. $AA_{aid}$ first chooses two random exponents $\alpha_{aid},\beta_{aid}\in Z_{p}$. For each attribute $x_{k}\in S_{A_{aid}}$, $AA_{aid}$ chooses an attribute version key as $VK_{x_{k}}=v_{x_{k}}$ and generates the public attribute keys as ${\left\{PK_{x_{k}}\right\}}_{aid\in I_{A}}=g_{2}^{v_{x_{k}}}\cdot g_{2}^{H(x_{k})}$. Then it publishes $PK_{aid}=e{\left(g_{1},g_{2}\right)}^{\alpha_{aid}}$ as its public key and keeps $SK_{aid}=\left\{\alpha_{aid},\beta_{aid}\right\}$ as its secret key.

*Encrypt_out* $(GP,{\left\{PK_{x_{k}}\right\}}_{aid\in I_{A}})\to CT_{out}$. FD first chooses a random number $s^{\prime }\in Z_{p}$ and computes $C_{0}=g_{1}^{s^{\prime }}$. For i∈{1,⋯,l}, it randomly picks ${\lambda^{\prime }}_{i},\gamma^{\prime }\in Z_{p}$ and computes:

(1) $$C_{i,1}=g_{2}{}^{a{\lambda^{\prime }}_{i}}\cdot {\left(g_{2}^{v_{x_{i}}}g_{2}^{H(x_{i})}\right)}^{-{\gamma^{\prime }}_{i}},C_{i,2}=g_{1}^{{\gamma^{\prime }}_{i}},s^{\prime },{\lambda^{\prime }}_{i},{\gamma^{\prime }}_{i}$$

Then, it outputs the partially encrypted ciphertext $CT_{out}=\left\{s^{\prime },C_{0},{\left(C_{i,1},C_{i,2},{\lambda^{\prime }}_{i},{\gamma^{\prime }}_{i}\right)}_{i\in \{1,\cdots ,l\}}\right\}$.

*Encrypt_user* $(GP,PK_{aid},{\left\{PK_{x_{k}}\right\}}_{aid\in I_{A}},CT_{out},M,(A,\rho ))\to CT$. Let A be a l×n matrix, where l denotes the total number of all the attributes. The function ρ maps rows of the matrix A to attributes. DO first chooses a random secret exponent $s\in Z_{p}$ and a random vector $\vec{v}=\left(s,y_{2},\cdots ,y_{n}\right)\in Z_{p}^{n}$ with s as its first entry, where $y_{2},\cdots ,y_{n}$ are used to share the secret exponent s. For i=1,⋯,l, it computes $\lambda_{i}=A_{i}\cdot \vec{v}$, where $A_{i}$ is the vector corresponding to the i-th row of A. After that, it randomly chooses $\gamma_{1},\gamma_{2},\cdots ,\gamma_{l}\in Z_{p}$ and computes:

(2) $$CT_{user}=\left\{\begin{array}{l} C=M\cdot e{\left(g_{1},g_{2}\right)}^{\sum_{aid\in I_{A}}\alpha_{aid}s},\;C^{\prime }=s-s^{\prime },\;{\left(C_{i,3}=\lambda_{i}-{\lambda^{\prime }}_{i},C_{i,4}=\gamma_{i}-{\gamma^{\prime }}_{i}\right)}_{i\in \{1,\cdots ,l\}},\\ C_{v}=G(e{\left(g_{1},g_{2}\right)}^{\sum_{aid\in I_{A}}\alpha_{aid}s}),\;(A,\rho ) \end{array}\right\}$$

$C^{\prime },C_{i,3},C_{i,4}$ are used to correct the shares of s and randomize $\gamma_{i}$. $C_{v}$ is used to verify the result of outsourced decryption. Then, it outputs the intact ciphertext $CT=\{C,C^{\prime },C_{0},{\left(C_{i,1},C_{i,2},C_{i,3},C_{i,4}\right)}_{i\in \{1,\cdots ,l\}},C_{v},(A,\rho )\}$.

*KeyGen* $(GP,uid,S_{uid,aid},SK_{aid},{\left\{PK_{x_{k}}\right\}}_{aid\in I_{A}})\to \left\{PxK_{uid,aid},SK_{uid}\right\}$. $AA_{aid}$ first assigns a set of attributes $S_{uid,aid}$ to each legal user, then chooses a random number $z_{uid}\in Z_{p}$ for each user and let $SK_{uid}=\left\{z_{uid}\right\}$ as the user secret key. Then, $AA_{aid}$ runs the key generation algorithm to generate the user proxy key as:

(3) $$PxK_{uid,aid}=\left\{K_{uid,aid}=g_{2}^{\frac{\alpha_{aid}}{z_{uid}}}g_{2}^{\frac{a\beta_{aid}}{z_{uid}}},L_{uid,aid}=g_{1}^{\frac{\beta_{aid}}{z_{uid}}},{\left\{K_{uid,x_{i}}\right\}}_{x_{i}\in S_{uid,aid}}={\left(g_{2}^{v_{x_{i}}}g_{2}^{H(x_{i})}\right)}^{\frac{\beta_{aid}}{z_{uid}}},S_{uid,aid}\right\}$$

The proxy keys $\left\{PxK_{uid,aid}\right\}$ are sent to CSP who will add them in its proxy key list $L_{PxK}$ as $L_{PxK}=L_{PxK}\cup \{uid,PxK_{uid,aid}\}$, and the user secret keys are sent to the corresponding DUs.

*Decrypt_out* $(GP,CT,PxK_{uid,aid},{\left\{PK_{x_{k}}\right\}}_{aid\in I_{A}})\to CT^{\prime }$. When a user queries the encrypted data in the system, CSP will first check his attribute set. If his attributes does not satisfy the access policy, CSP outputs ⊥. Otherwise, it sends the ciphertext and the corresponding proxy keys to FD. FD first chooses a set of constants $w_{i}\in Z_{p}$ such that, if $\lambda_{i}$ are valid shares of the secret s according to A, then $\sum_{i\in I}w_{i}\lambda_{i}=s$, where I={1,⋯,l}. Then it computes:

(4) $$CT^{\prime }=\prod_{aid\in I_{A}}\frac{e(C_{0}\cdot g_{1}^{C^{\prime }},K_{uid,aid})}{\prod_{i\in I}\left(e({\left(C_{i,1}\cdot g_{2}^{aC_{i,3}}\cdot {\left(g_{2}^{v_{x_{i}}}g_{2}^{H(x_{i})}\right)}^{-C_{i,4}}\right)}^{\omega_{i}},L_{uid,aid})\cdot e({\left(C_{i,2}\cdot g_{1}^{C_{i,4}}\right)}^{\omega_{i}},K_{uid,x_{i}})\right)}=e{\left(g_{1},g_{2}\right)}^{\sum_{aid\in I_{A}}\frac{\alpha_{aid}s}{z_{uid}}}$$

After that, FD sends the partially decrypted ciphertext $CT^{\prime }=e{\left(g_{1},g_{2}\right)}^{\sum_{k\in I_{A}}\frac{\alpha_{k}s}{z_{j}}}$ to the user.

*Decrypt_user* $(CT,CT^{\prime },SK_{uid})\to M$. Upon receiving the partially decrypted ciphertext from FD, the user runs the user decryption algorithm to decrypt the ciphertext by using its secret key $SK_{uid}$. It computes M as:

(5) $$M=\frac{C}{C{T^{\prime }}^{z_{uid}}}$$

### 4.2. Verification Method

There exists such a situation that the FD may be “lazy”. It may not follow the algorithm, only execute part of the computations or deliberately returns incorrect results. If this happens, a DO cannot notice the error, and large part of the computations will be affected. Therefore, we propose a verification method which can verify the result of outsourced encryption and outsourced decryption.

Our verification method includes two algorithms: 

*Verify_enc* $(GP,CT_{out})\to b$. Upon receiving the partially encrypted ciphertext $CT_{out}$ from FD, DO first verify whether $C_{0}=g^{s^{\prime }}$ holds. If it does not holds, DO outputs b=0, which indicates FD returns incorrect result. Otherwise, DO computes $t_{i}=(a{\lambda^{\prime }}_{i}-v_{\rho (i)}\cdot \gamma_{i}-H(\rho (i))\cdot \gamma_{i})\mathrm{mod}p$ and $C_{i}=C_{i,1}\cdot C_{i,2}=g_{1}^{{\gamma^{\prime }}_{i}}\cdot g_{2}^{t_{i}}$, where i∈{1,⋯,l}. Then, it picks a security parameter r, and randomly chooses $s_{1},\cdots ,s_{l}\in {\left\{0,1\right\}}^{r}$. After that, it computes $x=\sum_{i=1}^{n}{\gamma^{\prime }}_{i}s_{i}\mathrm{mod}p,y=\sum_{i=1}^{n}t_{i}s_{i}\mathrm{mod}p$ and $\hat{C}=\prod_{i=1}^{n}C_{i}^{s_{i}}\mathrm{mod}p$. If $\hat{C}=g_{1}^{x}\cdot g_{2}^{y}$, DO outputs b=1, which indicates the FD returned the correct result. Otherwise, it outputs b=0.

Here, we adopt the idea of [34]. We do not check the results in the ciphertext one by one. Instead, we use the batch verification algorithm to check $C_{i,1},C_{i,2}$ together. Obviously, our solution is much more efficient than the normal verification method.

*Verify_dec* $(GP,CT,CT^{\prime })\to b$. Upon receiving the partially decrypted ciphertext $CT^{\prime }$ from FD, the user computes $G(C{T^{\prime }}^{z_{uid}})$, if $G(C{T^{\prime }}^{z_{uid}})=C_{v}$ holds, it outputs b=1, which indicates FD returns the correct result. Otherwise, it outputs b=0, which indicates that the FD has returned an incorrect result.

### 4.3. Revocation Scheme

#### 4.3.1. User Revocation

In our scheme, when user revocation happens, we do not need to re-encrypt the ciphertext and update other non-revoked users’ secret keys. The only operation we need is to send a user revocation message which contains the uid of the revoked user to the CSP, and then let the CSP delete the revoked user’s proxy key $PxK_{uid,aid}$. Without the correct $PxK_{uid,aid}$, the FD cannot perform the outsourced decryption algorithm for the revoked user. Thus, the revoked user cannot recover the original data. The user revocation algorithm is described as follows:

*URev* $(uid,L_{PxK})\to {L^{\prime }}_{PxK}$. When the CSP receives the user revocation message from a DO, it then deletes the proxy key $PxK_{uid,aid}$ corresponding to the uid from the list and outputs the updated proxy key list ${L^{\prime }}_{PxK}$.

#### 4.3.2. Attribute Revocation

There are two phases in attribute revocation: Key update and Ciphertext re-encryption.

Phase 1: Key update

The key update in turn includes three steps: RekeyUpdate, CTUpdate and PxKUpdate.

(1) *ReKeyUpdate* $\left(uid,PxK_{uid,aid},v_{{\tilde{x}}_{k}})\to \{VUK_{{\tilde{x}}_{k}},PxUK_{{\tilde{x}}_{k}}\right\}$.

Let uid denotes all other non-revoked users except the revoked user with $uid^{\prime }$. The involved authority $AA_{aid}$ first generates a new attribute version key ${v^{\prime }}_{{\tilde{x}}_{k}}$. It then computes the version update key as $VUK_{{\tilde{x}}_{k}}={v^{\prime }}_{{\tilde{x}}_{k}}-v_{{\tilde{x}}_{k}}$. After that, it applies $VUK_{{\tilde{x}}_{k}}$ to compute the proxy update key as $PxUK_{{\tilde{x}}_{k}}=g_{2}^{\frac{\beta_{aid}}{z_{uid}}\cdot VUK_{{\tilde{x}}_{k}}}$ for each non-revoked user who has the attribute ${\tilde{x}}_{k}$. Then $AA_{aid}$ updates the public attribute key of the revoked attribute as $PK_{{\tilde{x}}_{k}}^{*}=PK_{{\tilde{x}}_{k}}\cdot g_{2}^{VUK_{{\tilde{x}}_{k}}}$, and broadcast a message for each DO such that they can get the updated public attribute key of the revoked attribute. After that, $PxUK_{{\tilde{x}}_{k}}$ is sent to the CSP to update $PxK_{uid,aid}$ and $VUK_{{\tilde{x}}_{k}}$ is sent to the DO.

(2) *CTUpdate* $\left(VUK_{{\tilde{x}}_{k}},CT_{out})\to \{CUK_{{\tilde{x}}_{k}}\right\}$. 

Upon receiving the version update key $VUK_{{\tilde{x}}_{k}}$ and the partially encrypted ciphertext $CT_{out}$, DO computes the ciphertext update key as $CUK_{{\tilde{x}}_{k}}=g_{2}^{{}^{-{\gamma^{\prime }}_{i}\cdot AUK_{{\tilde{x}}_{k}}}}$. Then, $CUK_{{\tilde{x}}_{k}}$ is sent to the CSP to update the ciphertext.

(3) *PxKUpdate* $(uid,PxK_{uid,aid},PxUK_{{\tilde{x}}_{k}})\to PxK_{uid,aid}^{*}$. 

Upon receiving the proxy update key $PxUK_{{\tilde{x}}_{k}}$, CSP updates the corresponding proxy keys as $K_{x={\tilde{x}}_{k},uid}^{*}=K_{x={\tilde{x}}_{k},uid}\cdot PxUK_{{\tilde{x}}_{k}}$ for each non-revoked user who has the attribute ${\tilde{x}}_{k}$. Then the proxy keys $PxK_{uid,aid}$ are updated as:

(6) $$PxK_{uid,aid}^{*}=\left\{\begin{array}{l} K_{uid,aid}=g_{2}^{\frac{\alpha_{aid}}{z_{uid}}}g_{2}^{\frac{a\beta_{aid}}{z_{uid}}},L_{uid,aid}=g_{1}^{\frac{\beta_{aid}}{z_{uid}}}\\ K_{uid,x\neq {\tilde{x}}_{k}}={\left(g_{2}^{v_{x}}g_{2}^{H(x)}\right)}^{\frac{\beta_{aid}}{z_{uid}}}\\ K_{uid,x={\tilde{x}}_{k}}^{*}={\left(g_{2}^{{v^{\prime }}_{{\tilde{x}}_{k}}}g_{2}^{H({\tilde{x}}_{k})}\right)}^{\frac{\beta_{aid}}{z_{uid}}} \end{array}\right\}$$

Phase 2: Ciphertext re-encryption

*ReEnc* $(CT,CUK_{{\tilde{x}}_{k}})\to CT^{*}$. 

Upon receiving the ciphertext update key $CUK_{{\tilde{x}}_{k}}$, CSP updates the corresponding ciphertext as $C_{i,1}^{*}=C_{i,1}\cdot CUK_{{\tilde{x}}_{k}}$. Then the new ciphertext $CT^{*}$ is published as:

(7) $$CT^{*}=\left\{\begin{array}{l} C,C^{\prime },C_{0},{\left(C_{i,2},C_{i,3},C_{i,4}\right)}_{i\in \{1,\cdots ,l\}},C_{v},(A,\rho )\\ {\left(C_{i,1}=g_{2}{}^{a{\lambda^{\prime }}_{i}}\cdot {\left(g_{2}^{v_{x_{i}}}g_{2}^{H(x_{i})}\right)}^{-{\gamma^{\prime }}_{i}}\right)}_{x_{i}\neq {\tilde{x}}_{k},i\in \{1,\cdots ,l\}}\\ {\left(C_{i,1}^{*}=g_{2}{}^{a{\lambda^{\prime }}_{i}}\cdot {\left(g_{2}^{{v^{\prime }}_{x_{k}}}g_{2}^{H(x_{k})}\right)}^{-{\gamma^{\prime }}_{i}}\right)}_{x_{i}={\tilde{x}}_{k},i\in \{1,\cdots ,l\}} \end{array}\right\}$$

Apparently, we can conclude that most of the update and re-encryption work is outsourced to the CSP, which greatly reduces the overhead of DOs. Meanwhile, we do not need to update the entire ciphertext and user proxy keys. Only those components which are involved with the revoked attribute need to be updated. In this way, our scheme can greatly improve the efficiency of attribute revocation.

## 5. Analysis of Our Scheme

In this section, a comprehensive analysis of VO-MAACS is provided, including security analysis and performance analysis.

### 5.1. Security Analysis

#### 5.1.1. Correctness

The correctness of our scheme can be easily proved by the following equations:

When there is no attribute revocation:

$$\begin{array}{ll} CT^{\prime } & =\prod_{aid\in I_{A}}\frac{e(C_{0}\cdot g_{1}^{C^{\prime }},K_{uid,aid})}{\prod_{i\in I}\left(e({\left(C_{i,1}\cdot g_{2}^{aC_{i,3}}\cdot {\left(g_{2}^{v_{x_{i}}}g_{2}^{H(x_{i})}\right)}^{-C_{i,4}}\right)}^{\omega_{i}},L_{uid,aid})\cdot e({\left(C_{i,2}\cdot g_{1}^{C_{i,4}}\right)}^{\omega_{i}},K_{uid,x_{i}})\right)}\\ & =\prod_{aid\in I_{A}}\frac{e(g_{1}^{s},g_{2}^{\frac{\alpha_{aid}}{z_{uid}}}g_{2}^{\frac{a\beta_{aid}}{z_{uid}}})}{\prod_{i\in I}\left(e((g_{2}{}^{a\lambda_{i}\omega_{i}}\cdot {\left(g_{2}^{v_{x_{i}}}g_{2}^{H(x_{i})}\right)}^{-\gamma_{i}\omega_{i}},g_{1}^{\frac{\beta_{aid}}{z_{uid}}})\cdot e(g_{1}^{\gamma_{i}\omega_{i}},{\left(g_{2}^{v_{x_{i}}}g_{2}^{H(x_{i})}\right)}^{\frac{\beta_{aid}}{z_{uid}}})\right)}\\ & =e{\left(g_{1},g_{2}\right)}^{\sum_{aid\in I_{A}}\frac{\alpha_{aid}s}{z_{uid}}} \end{array}$$

When the attribute ${\tilde{x}}_{k}$ is revoked from a user whose identity is uid:

For $x_{i}\neq {\tilde{x}}_{k}$:

$$\begin{array}{ll} CT_{i} & =\frac{e(C_{0}\cdot g_{1}^{C^{\prime }},K_{uid,aid})}{\left(e({\left(C_{i,1}\cdot g_{2}^{aC_{i,3}}\cdot {\left(g_{2}^{v_{x_{i}}}g_{2}^{H(x_{i})}\right)}^{-C_{i,4}}\right)}^{\omega_{i}},L_{uid,aid})\cdot e({\left(C_{i,2}\cdot g_{1}^{C_{i,4}}\right)}^{\omega_{i}},K_{uid,x_{i}})\right)}\\ & =\frac{e(g_{1}^{s},g_{2}^{\frac{\alpha_{aid}}{z_{uid}}}g_{2}^{\frac{a\beta_{aid}}{z_{uid}}})}{e((g_{2}{}^{a\lambda_{i}\omega_{i}}\cdot {\left(g_{2}^{v_{x_{i}}}g_{2}^{H(x_{i})}\right)}^{-\gamma_{i}\omega_{i}},g_{1}^{\frac{\beta_{aid}}{z_{uid}}})\cdot e(g_{1}^{-\gamma_{i}\omega_{i}},{\left(g_{2}^{v_{x_{i}}}g_{2}^{H(x_{i})}\right)}^{\frac{\beta_{aid}}{z_{uid}}})}\\ & =e{\left(g_{1},g_{2}\right)}^{\frac{\alpha_{aid}s}{z_{uid}}} \end{array}$$

For $x_{i}={\tilde{x}}_{k}$:

$$\begin{array}{ll} CT_{i} & =\frac{e(C_{0}\cdot g_{1}^{C^{\prime }},K_{uid,aid})}{\left(e({\left(C_{i,1}\cdot g_{2}^{aC_{i,3}}\cdot {\left(g_{2}^{{v^{\prime }}_{x_{i}}}g_{2}^{H(x_{i})}\right)}^{-C_{i,4}}\right)}^{\omega_{i}},L_{uid,aid})\cdot e({\left(C_{i,2}\cdot g_{1}^{C_{i,4}}\right)}^{\omega_{i}},K_{uid,x_{i}})\right)}\\ & =\frac{e(g_{1}^{s},g_{2}^{\frac{\alpha_{aid}}{z_{uid}}}g_{2}^{\frac{a\beta_{aid}}{z_{uid}}})}{e((g_{2}{}^{a\lambda_{i}\omega_{i}}\cdot {\left(g_{2}^{{v^{\prime }}_{x_{i}}}g_{2}^{H(x_{i})}\right)}^{-\gamma_{i}\omega_{i}},g_{1}^{\frac{\beta_{aid}}{z_{uid}}})\cdot e(g_{1}^{\gamma_{i}\omega_{i}},{\left(g_{2}^{{v^{\prime }}_{x_{i}}}g_{2}^{H(x_{i})}\right)}^{\frac{\beta_{aid}}{z_{uid}}})}\\ & =e{\left(g_{1},g_{2}\right)}^{\frac{\alpha_{aid}s}{z_{uid}}} \end{array}$$

Therefore:

$$CT^{\prime }=\prod_{aid\in I_{A}}CT_{i}=\prod_{aid\in I_{A}}e{\left(g_{1},g_{2}\right)}^{\frac{\alpha_{aid}s}{z_{uid}}}=e{\left(g_{1},g_{2}\right)}^{\sum_{aid\in I_{A}}\frac{\alpha_{aid}s}{z_{uid}}}$$

Then:

$$\frac{C}{C{T^{\prime }}^{z_{uid}}}=\frac{M\cdot e{\left(g_{1},g_{2}\right)}^{\sum_{aid\in I_{A}}\alpha_{aid}s}}{e{\left(g_{1},g_{2}\right)}^{\sum_{aid\in I_{A}}\alpha_{aid}s}}=M$$

Therefore, VO-MAACS satisfies correctness.

#### 5.1.2. Data Confidentiality

In our system, only for users whose attributes satisfy the access policy, will the FD decrypt the ciphertext for them by using their proxy keys. Users whose attributes do not satisfy the access policy, cannot receive the partially decrypted ciphertext from the FD. Thus, they are not able to recover the original data. When a user is revoked, his proxy key will be deleted by the CSP. Without the proxy key, he cannot obtain the partially decrypted ciphertext either. Therefore, for users whose attributes do not satisfy the access policy, our solution satisfies the data confidentiality.

In addition, although the CSP and FD can get user proxy keys, however, if they do not obtain the user secret keys, they still cannot decrypt the ciphertext. Similarly, they cannot collude with other users to recover the data either. Therefore, for the CSP and FD, our solution also satisfies the data confidentiality.

#### 5.1.3. Collusion tolerance

In our system, each user is assigned with a unique identity *uid*, and each key issued by different AA is associated with a *uid*. Therefore, only the keys associated with the same *uid* can be used to decrypt the ciphertext. Other users cannot collude to decrypt the ciphertext. In addition, there exists a situation that some AAs may issue the same attributes. Since each AA has a unique identity *aid*, all attributes are distinguishable. Therefore, users cannot replace some of the components in the keys from the AA by using the component in the key from another AA.

### 5.2. Performance Analysis

We implement our scheme in Charm [35], a framework developed to facilitate the rapid prototyping of cryptographic schemes and protocols. It is based on the Python language which allows the programmer to write code similar to the theoretical implementations. Charm also provides routines for applying and using LSSS schemes needed for Attribute-Based systems. All our implementations are executed on an Intel^®^ Pentium^®^ CPU G630@270 GHz with 4.00 GB RAM running Ubuntu14.04 64-bit system and Python 2.7.

**Figure 3:**
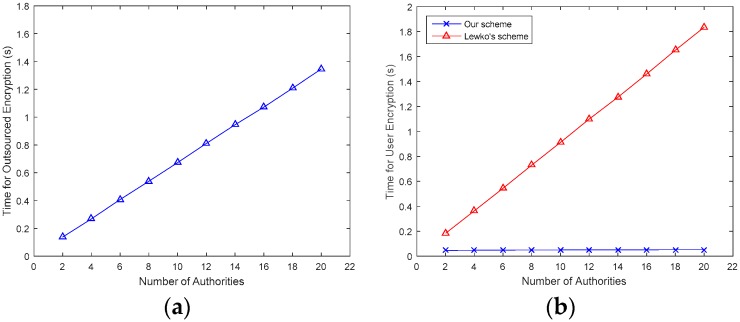
Comparison of encryption time with different number of authorities. (**a**) Encrypt_out time; (**b**) Encrypt_user time.

In our experiment, access policies are generated in the form of *a*_1_, *a*_2_, …, *a_n_*, where *a_i_* is an attribute. We set 20 distinct access policies in this form with N increasing from 20 to 200, and repeat each instance 20 times and take the average values as the experiment results. We simulate the computing time incurred in encryption and decryption. Since our scheme is based on Lewko’s scheme [14], we compare our scheme with [14] in user encryption time and user decryption time. In our experiments, the number of attributes per authority is set to 10. The times for outsourced encryption are shown in Figure 3a,b, respectively. 

**Figure 4:**
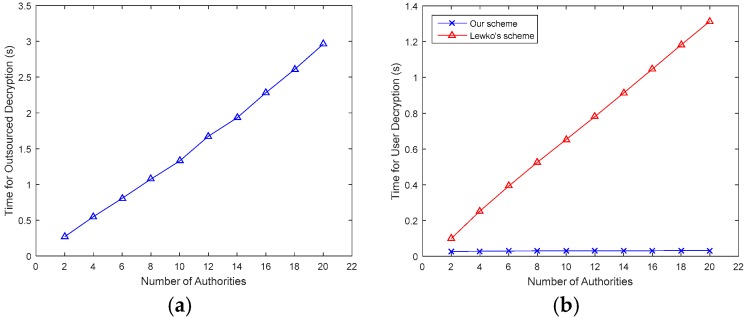
Comparison of decryption time with different number of authorities. (**a**) Decrypt_out time; (**b**) Decrypt_user time.

In Figure 3a, the Encrypt_out time is approximately 0.1~1.4 s, and it increases almost linearly with the number of attributes. In Figure 3b, since major computations are outsourced to the FDs, only a few operations are left for DOs. Therefore, the Encrypt_user time in our scheme is much less than that in [14]. Similarly, Figure 4 describes the time for outsourced decryption and user decryption. In Figure 4a, the Decrypt_out time is approximately 0.3~3 s, and like the Encrpyt_time, it also increases linearly with the number of attributes. In Figure 4b, as major computations are outsourced to FDs, only a few operations are left for DUs, therefore, the Decrypt_user time in our scheme is much less than that in [14].

**Figure 5:**
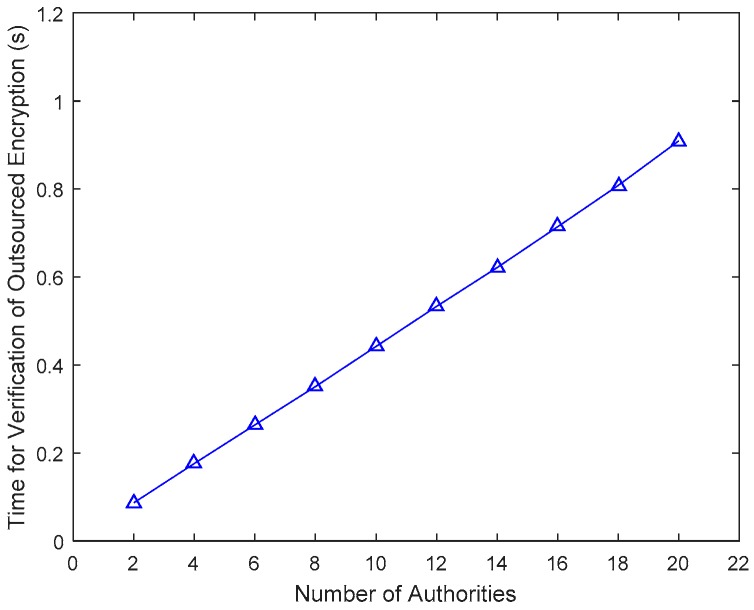
Computing cost for verification of outsourced encryption.

**Figure 6:**
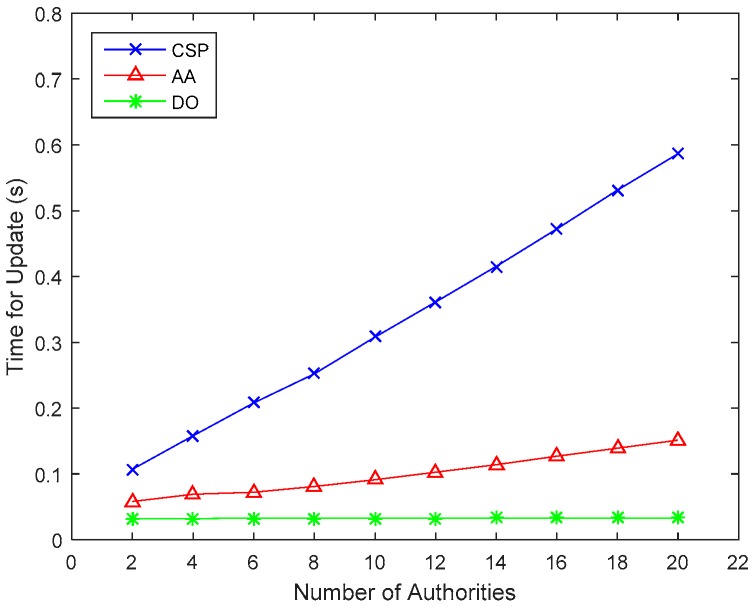
Comparison of computing cost of CSP, AA and DO in the attribute revocation process.

The computing cost for verification of outsourced encryption is shown in Figure 5. The time for Verify_enc is approximately 0.1~0.8 s and it increases almost linearly with the number of attributes. Figure 6 describes the comparison of computing cost of CSP, AA and DO in the attribute revocation process. 

In fact, most computing overhead, such as proxy keys update and ciphertext re-encryption are outsourced to the CSP, and only a few computations are left for AAs and DOs. Therefore, the computing cost for DOs can be greatly reduced. Apparently, our scheme requires less time for both encryption and decryption than Lewko’s scheme, and the computing cost for DOs in the attribute revocation process is greatly reduced. Therefore, we can conclude that our scheme’s computation efficiency is much better than that of Lewko’s scheme.

## 6. Conclusions

To realize data access control in fog-cloud computing system, we have proposed a verifiable outsourced multi-authority access control scheme, named VO-MAACS. In our construct, most encryption and decryption computations are outsourced to fog devices and the computation results can be verified by using our verification method. Meanwhile, to address the revocation issue, we designed an efficient user and attribute revocation method for this. Finally, the analysis and simulation results show that our scheme is both secure and highly efficient.
